# A Study on the Correlation Between Age-Related Macular Degeneration and Alzheimer's Disease Based on the Application of Artificial Neural Network

**DOI:** 10.3389/fpubh.2022.925147

**Published:** 2022-06-30

**Authors:** Meng Zhang, Xuewu Gong, Wenhui Ma, Libo Wen, Yuejing Wang, Hongbo Yao

**Affiliations:** ^1^Histology and Embryology Section, Qiqihar Medical University, Qiqihar, China; ^2^The Second Affiliated Hospital of Qiqihar Medical University, Qiqihar, China; ^3^Computer Experimental Teaching Center, Qiqihar Medical University, Qiqihar, China; ^4^Physiology Section, Qiqihar Medical University, Qiqihar, China

**Keywords:** artificial neural network, age-related macular degeneration, Alzheimer's disease, relevance, correlation

## Abstract

Age-related Macular Degeneration (AMD) is a kind of irreversible vision loss or disease caused by retinal pigment epithelial cells and neuroretinal degeneration, which has become the main cause of vision loss and blindness of the elderly over 65 years old in developed countries. The main clinical manifestations are cognitive decline, mental symptoms and behavioral disorders, and the gradual decline of daily living ability. In this paper, a feature extraction method of electroencephalogram (EEG) signal based on multi-spectral image fusion of multi-brain regions is proposed based on artificial neural network (ANN). In this method, the brain is divided into several different brain regions, and the EEG signals of different brain regions are transformed into several multispectral images by combining with the multispectral image transformation method. Using Alzheimer's disease (AD) classification algorithm, the depth residual network model pre-trained in ImageNet was transferred to sMRI data set for fine adjustment, instead of training a brand-new model from scratch. The results show that the proposed method solves the problem of few available medical image samples and shortens the training time of ANN model.

## Introduction

Alzheimer's disease (AD) is a kind of neurodegenerative disease, which is mainly characterized by memory disorder, cognitive disorder and language disorder, and is the most common cause of senile dementia (1). The latest survey report released by the International Association of AD shows that a new AD case will appear every 3 s around the world. The exact etiology and pathogenesis of AD are not clear, so there is no complete cure for AD. Once suffering from AD, it will cause destructive damage to the physiological structure of the brain. For example, various brain regions will gradually shrink. Because this injury is irreversible, the life expectancy of patients diagnosed with AD is only about 7 years (2). Therefore, how to control the progress of AD is extremely important.

Age-related macular degeneration (AMD) is the main cause of blindness in the elderly, especially irreversible visual loss. Cognitive function is an important part of the higher cerebral cortex, which is composed of memory, attention, calculation, orientation, executive ability, and so on. AD is the most common disease type of cognitive dysfunction. The prevalence of AMD and cognitive dysfunction increases with age, which has a great impact on people's life, study and work (3, 4). Literature (5) found that the score of the simple mental state examination scale in AMD group was lower than that of the matched control group without AMD, such as age, sex, and years of education, and the prevalence of AD in AMD patients was higher than that in the control group (40.7 vs. 20.4%, *p* = 0.03). Literature (6) a large-scale prospective study of AD, AMD, and control group showed that there was no significant difference between AD group and control group. Some studies have found that AMD and AD are not only common in epidemiology, but also common in molecular aspects (7–10). It is speculated that oxidative stress may be the earliest feature of AD, and the level of heme oxygenase-1, a sensitive marker of oxidative stress, was found in the brain tissues of AD and patients with mild cognitive impairment. Literature (11) found that aggregated Aβ is not only the activator of complement system in brain, but also the activator of microglia, which leads to the increase of oxidative stress. It has been found that microglia activation also exists around drusen and subretinal space, causing damage to brain and eyes. Literature (12, 13) in a population-based prospective cohort study, it was found that the newly diagnosed dry and wet AMD group was more likely to suffer from AD or Alzheimer's disease (*p* = 0.044) than the control group with no AMD at all and matched age, sex, and entry time. Compared with wet AMD patients, the relationship between dry AMD patients and AD is more obvious.

In the deep learning algorithm, convolutional neural network and deep neural network are used to classify electroencephalogram (EEG) signals of AMD and NC. Literature (14) classifies resting EEG signals of AD patients, patients with mild cognitive impairment and normal people by combining convolutional neural network with multilayer feed-forward perceptron, and finally obtains the best accuracy of 85% for the second classification and 82% for the third classification. Literature (15) classified the resting EEG signals of patients with AD, patients with mild cognitive impairment, and normal people by using convolutional neural network, and finally got the best accuracy of 89.8% for the second classification and 83.3% for the third classification (16). In the visual working memory paradigm, features are extracted from patients' task EEG signals, and then multi-kernel SVM is used for classification. Finally, the maximum classification accuracy is 91.76 and 82.23%, respectively, under the stimulus of 0-back and 1-back tasks.

Artificial neural network (ANN) is a complex network system, which consists of a large number of simple basic elements-neurons connected with each other, and performs information parallel processing and non-linear transformation by simulating the way of human brain nerve processing information (17). ANN can simulate many basic functions and ways of thinking of the brain, and acquire external knowledge through learning and store it in the network. ANN has been widely used in economy, engineering, medicine, biology, and other fields, and has become a unique information processing discipline. Compared with digital computers, ANN is closer to the human brain in terms of composition principle and functional characteristics. “Instead of executing operations step by step according to a given program, ANN can adapt itself to the environment and summarize laws!” Complete some operation, identification or process control (18). Based on the basic theory of ANN, this paper proposes an AD classification algorithm based on transfer learning and deep residual network. Migrate the pre-trained depth residual network model in ImageNet to sMRI data set for fine-tuning, instead of training a brand-new model from scratch; The results show that the proposed method solves the problem of few available medical image samples.

## Correlation Mechanism Between AMD and AD

Although AMD and AD are degenerative diseases of different tissues, because retina is an integral part of central nervous system, there may be a correlation between the pathology and pathogenesis of these two diseases.

### Pathophysiological Research

Some studies have found that AMD and AD are not only common in epidemiology, but also common in molecular aspects (19). For example, the characteristic senile plaque of AD and the marker drusen of AMD have common active ingredients, the most important of which is Aβ, both of which contain and (20).

In addition, a study showed that the defects of electroretinogram were eliminated in a dose-dependent manner by administering antibodies targeting and terminals to mice. The decrease of Aβ level in retinal pigment epithelial deposits and the preservation of retinal pigment epithelial structure were related to anti-q antibody immunotherapy and visual protection. These observations are consistent with the decrease of amyloid and the improvement of cognitive function in AD mice treated with anti-Aβ antibody (21).

### Relevance Mechanism

#### Chronic Inflammatory Reaction

Inflammatory reaction is the rapid response of cells to danger, with the aim of initiating immune response. In the short term, inflammatory reaction is beneficial, but a long-term chronic inflammatory reaction is harmful to the body. Long-term inflammation is related to the development of various chronic diseases, such as autoimmune diseases and neurodegenerative diseases (22). Amyloid protein and lipofuscin increase with age in normal eyes and brain, which activate inflammasome, complement system and autophagy lysosome and accelerate the progress of diseases (23). The studies have found that microglia activation also exists around drusen and subretinal space (24), which causes damage to brain and eyes. These phenomena are both the causes and consequences of Aβ formation. In this way, an irreversible positive feedback mechanism of pro-inflammatory cytokines and protease secretion occurs, which drives the progress of the disease (25).

#### Oxidative Stress

In the brain and eyes, the function of physiological cells needs oxidative stress, but when it exceeds a certain threshold, it will be cytotoxic. However, the human body has an antioxidant system to rebuild the internal balance: the dysfunction of this system may contribute to AD pathophysiology. It is speculated that oxidative stress may be the earliest feature of AD, and the level of heme oxygenase-1, a sensitive marker of oxidative stress, was found in the brain tissues of AD and patients with mild cognitive impairment. It has also been found that autophagy and lysosomes also participate in the oxidative stress process in the eyes of patients with AMD and the brains of patients with AD (26). In a word, excessive oxidative stress and dysfunction of mitochondria and lysosomes seem to be common pathophysiology in the pathogenesis of AMD and AD.

## Research Method

### Artificial Neural Network

Neural network is composed of a large number of neurons connected with each other. The processing ability of a single neuron is not strong, but after a large number of neurons are interconnected into a network, its processing ability is greatly improved. Neural network is a model obtained by abstracting and simplifying biological neural network on the basis of simulating the basic characteristics of brain. In neural networks, the storage of knowledge and information is characterized by the interconnected and distributed physical connections among neurons, while learning and recognition are the dynamic changes of the weighting coefficients of connections among neurons.

**Figure 1:**
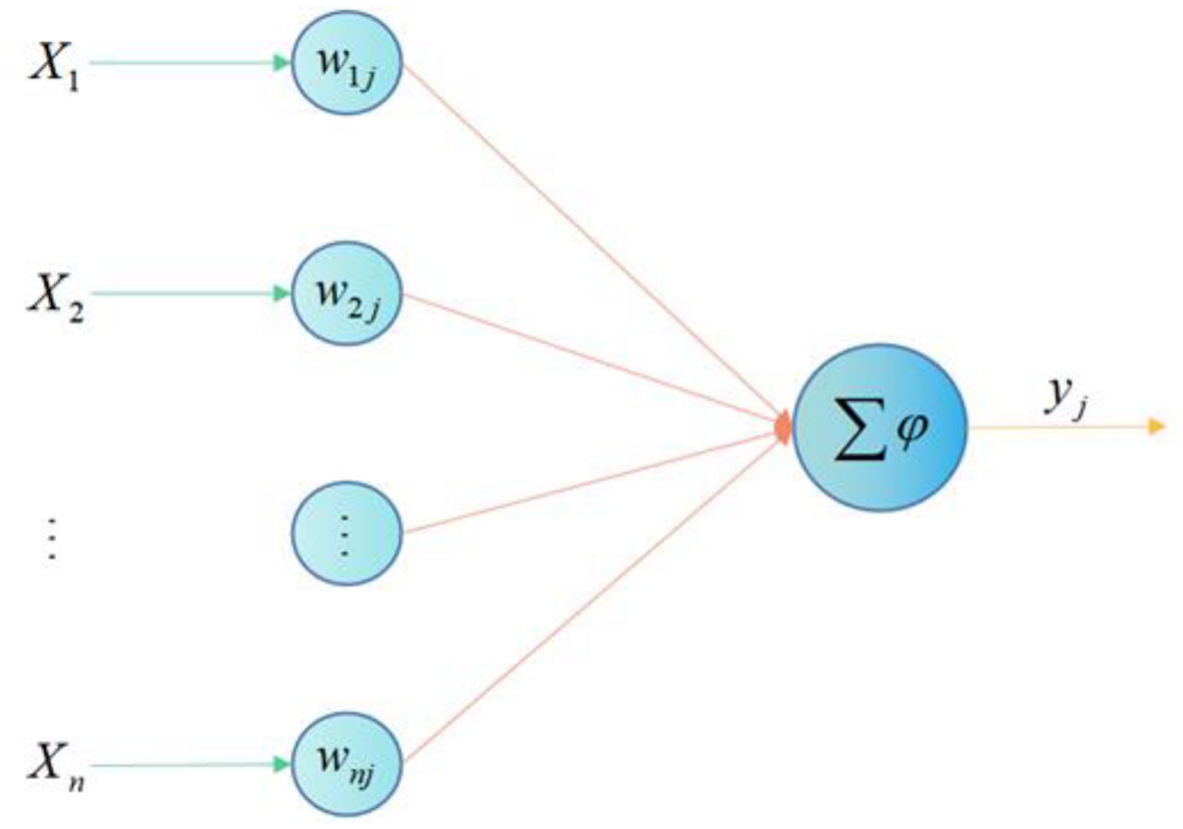
Neuron model.

It is the basic information processing unit of ANN operation, and the neuron model contains 3 basic elements: synapse, adder and activation function. Figure 1 shows the neuron model, where the input is signal and represents the synaptic weight of neuron.

The neuron in Figure 1 can be described by the following equations:


(1) $$\begin{array}{l} u_{j}=\overset{n}{\underset{i=1}{\sum }}\left(w_{nj}x_{n}\right) \end{array}$$

(2) $$\begin{array}{l} y_{j}=\phi \left(u_{j}+b_{j}\right) \end{array}$$

At present, according to different problems, different ANN models are proposed, and each algorithm is different. Common network models include BP neural network model, Hofield neural network model, linear neural network model, and Kohonen self-organizing model. These ANNs are not suitable for all the problems, so we must choose the appropriate ANNs according to the different problems to be solved. BP neural network and radial basis function neural network are often used in medical diagnosis and prediction, and perceptron model is mainly used for classification in clinic.

### Multi-Brain Multispectral Image Fusion Algorithm

A large number of studies have shown that there are differences in EEG signals between AMD and Normal Control (NC), which are generally manifested in five brain regions. The five brain regions are frontal lobe, parietal lobe, left temporal lobe, right temporal lobe, and occipital lobe (27). For example, in frontal lobe, there are differences between theta frequency band and delta frequency band between AMD and NC group. In parietal lobe, AMD and NC have different phase coupling in the cross-frequency band between alpha frequency band and beta frequency band. In this study, EEG signals of five brain regions were converted into multispectral images by multispectral image conversion method. Multispectral images representing different brain regions are obtained by using the method of multispectral image transformation alone in five brain regions.

In this study, the EEG signals of five brain regions were transformed into five multispectral images by the method of multispectral image conversion. Each brain region contains more electrodes, and if all the electrodes are used, the amount of calculation will be greatly increased. Therefore, some electrodes are selected from each brain region, and the EEG signals of these electrodes are converted into multispectral images.

In this study, pixel-level weighted average image fusion algorithm is used to fuse multispectral images of multiple brain regions. The fusion formula of the weighted average fusion method is shown in formula (3):


(3) $$\begin{array}{l} Y=\underset{n}{\sum }A_{n}X_{n} \end{array}$$

In formula (3), *A*_*n*_ represents the corresponding weight, $\underset{n}{\sum }A_{n}=1$, *X*_*n*_ represents the corresponding pixel, and *Y* represents the generated new pixel.

Because the multispectral image formed in this study is a 40^*^40^*^3 color image, each pixel *X* is composed of 3 values (RGB 3 primary colors), namely *X* = (*X*_*R*_, *X*_*G*_, *X*_*B*_). According to the above weighted average fusion formula, the following formula is obtained.


(4) $$\begin{array}{l} Y_{R}=\underset{n}{\sum }A_{n}X_{R}\\ Y_{G}=\underset{n}{\sum }A_{n}X_{G}\\ Y_{B}=\underset{n}{\sum }A_{n}X_{B} \end{array}$$

That is, for the pixels in each image, the corresponding R, G, and B are taken for weighted average fusion, and finally the corresponding pixels of the new multispectral image are generated.


(5) $$\begin{array}{l} Y=\left(\underset{n}{\sum }A_{n}X_{R},\underset{n}{\sum }A_{n}X_{G},\underset{n}{\sum }A_{n}X_{B}\right) \end{array}$$

**Figure 2:**
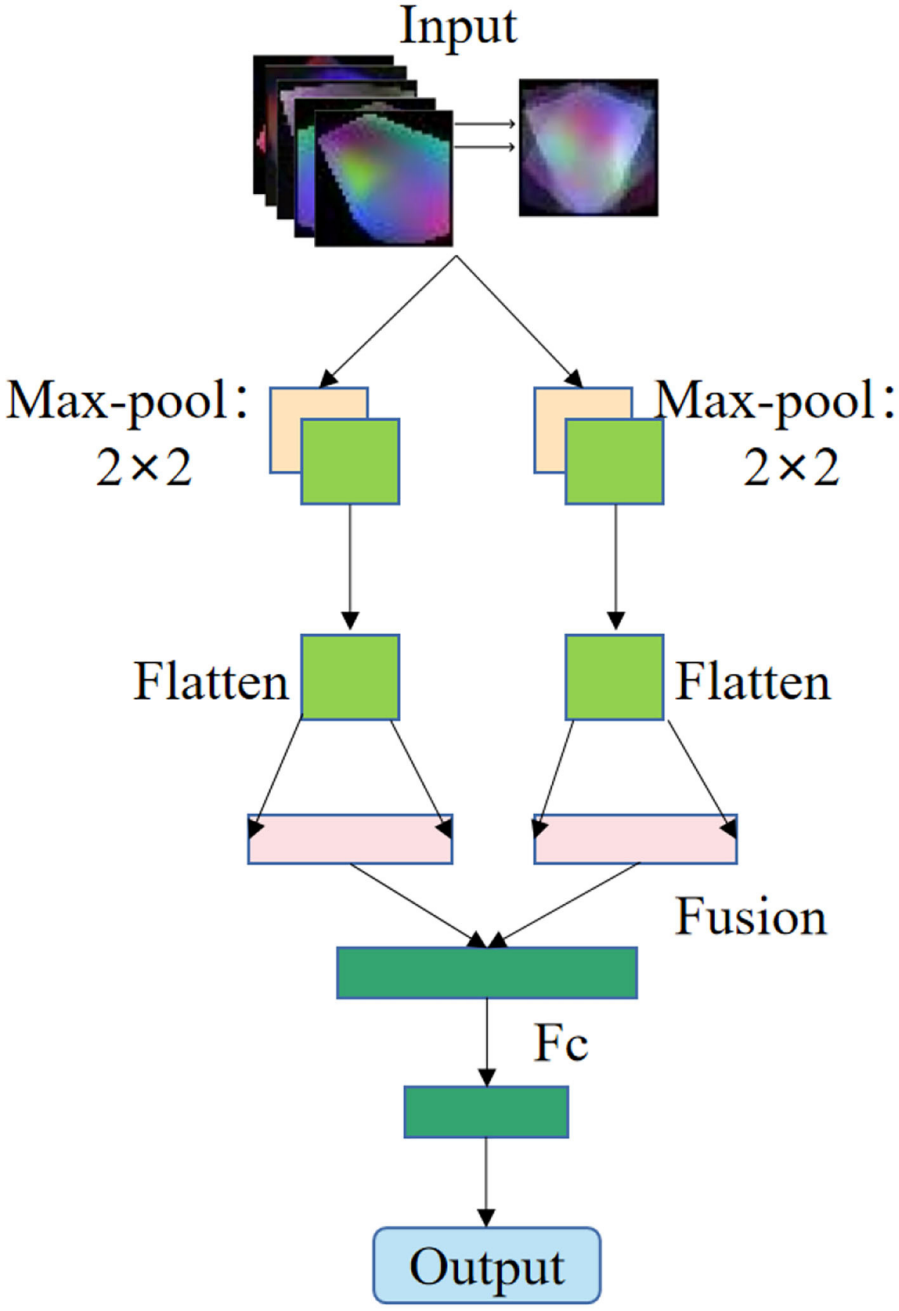
Schematic diagram of multispectral image fusion in multiple brain regions.

In this study, the pixel-level weighted fusion method of image fusion algorithm is used to fuse these 5 images to form a new image, as shown in Figure 2.

The feature extraction method of EEG signal based on multispectral image fusion of brain regions is a combination of multispectral image transformation method and image fusion method. Firstly, the EEG signals of each brain region are extracted according to the electrode position (28). Secondly, the multi-spectral image transformation method is used to transform the EEG signals of each brain region into multi-spectral images of the characteristics of each brain region. Finally, the image weighted average fusion algorithm is used to fuse the multispectral images of the five brain regions to form the fused multispectral image as shown in Figure 2.

Multi-brain multispectral image fusion;

Input: EEG of task EEG.

Output: multispectral images of multiple brain regions and fused multispectral images.

BEGIN

(1) *X*_*i*_ = *EEG*(*i*). //The electrode signals of the 5 selected brain regions are extracted, in which the values of *i* are e, d, ZN, YN, and z, which respectively represent frontal lobe, parietal lobe, left temporal lobe, right temporal lobe and occipital lobe.
(2) *Y*_*i*_ = *FFT*(*X*_*i*_). //Fourier transform was carried out on EEG signals of each brain region.
(3) *Z*_*j*_ = *Y*_*i*_(*j*). //The values of *j* are alpha, beta, and theta, which means extracting signals of 3 frequency bands.
(4) *Z*_*j*_ = *square*(*Z*_*j*_). //Take the sum of squares for the signals of the three frequency bands respectively.
(5) Local_2d = AEP(Local_3d). //According to AEP method, the electrode 3d coordinates are converted into 2d coordinates.
(6) *D*_*i*_ = *Conver*_*to*_*image*(*Z*_*theta*_, *Z*_*theta*_, *Z*_*beta*_ = *R, G, B*|Local_2d, Clough-Tocher). //The features of the sum of squares of the three frequency bands of each electrode are transformed into images according to the 2d coordinates of the electrodes, and the surrounding pixels are filled up by Clough-Tocher interpolation method. The images formed by the three frequency bands are used as RGB channels of color images, and a color multispectral image is formed.
(7) *Output*(*D*_*i*_). //Outputting multispectral images of five brain regions.
(8) $D=\underset{i}{\sum }W_{i}D_{i}$. //Image-level weighted average fusion method, that is, adding corresponding pixels, in which the weight sum is 1, that is, $\underset{i}{\sum }W_{i}=1$.
(9) *Output*(*D*). //Output a new multispectral image after fusion.

END

### Group Diagram Construction

The key to construct population map is to select the phenotypic measure that best explains the similarity between imaging data or the similarity between subject tags. In the ANN method, there are *h* sets of phenotypic information *M* = {*M*_*h*_} (such as gender and age of the subjects), and the adjacency matrix*W* of the population graph is defined as:


(6) $$\begin{array}{l} W\left(v,w\right)=sim\left(A_{v},A_{w}\right)\overset{H}{\underset{h=1}{\sum }}\gamma \left(M_{h}\left(v\right),M_{h}\left(w\right)\right) \end{array}$$

This formula represents the edge weight between the tested *v* and the tested *w*, which consists of 2 parts: *sim*(*A*_*v*_, *A*_*w*_) represents the similarity of brain network features between the tested, and increases the edge weight between the most similar nodes; γ represents the similarity between phenotypic information.

Specifically, feature similarity is defined as:


(7) $$\begin{array}{l} sim\left(A_{v},A_{w}\right)=\exp\left(-\frac{{\left(\rho \left(x\left(v\right),x\left(v\right)\right)\right)}^{2}}{2\sigma^{2}}\right) \end{array}$$

Where, ρ(*x*(*v*), *x*(*v*)) ∈ (0, 1) is the similarity distance between two subjects, and the more similar the two groups are, the closer the similarity distance is to 1; σ is the kernel width, so the neighborhood range can be customized.

The idea of this feature similarity measure is that subjects belonging to the same class (healthy or ill) have more similar networks than subjects from different classes, that is, larger sim values. The definition of phenotypic information similarity can be divided into quantitative type and qualitative type (29). For qualitative phenotypic information with gender as an example, the Kroneckerdelta formula is used to defineγ. For example, if two subjects have the same gender, the weight of the edge between two nodes will increase.

For quantitative phenotypic information with age as an example, if the difference between them is less than the present value, it is considered to be similar, and the edge weight between two nodes is set to 1; otherwise, it is considered that the information is not similar, and the edge weight is 0. γ is defined by a unit step function with threshold θ, which includes:


(8) $$\gamma \left(M_{h}\left(v\right),M_{h}\left(w\right)\right)=\{\begin{array}{c} 1,\;\left(\left|M_{h}\left(v\right),M_{h}\left(w\right)\right|<\theta \right)\\ 0,\text{ Otherwise} \end{array}$$

Finally, the similarity of eigenvalues is multiplied by the similarity of phenotypic information, which is integrated into the total edge weight to obtain the final group adjacency matrix, that is, the graph structure. This definition of similarity reflects strong similarity in the same test.

### AD Classification Algorithm

The Residual Neural Network (ResNet) was proposed by He et al. (30). The depth ResNet consists of a group of residual blocks, each of which consists of convolution layer, batch normalization layer and active function rectification linear unit. ResNets can be regarded as multiple basic blocks connected in series with each other. This shortcut can skip each basic block in parallel and then connect it to the output of the stack layer. It is easier to optimize the residual mapping than to optimize the original, unreferenced mapping.

Based on the special design of deep ResNet, the problems such as gradient explosion and gradient disappearance are effectively suppressed, so that the network can be fully trained, the number of network layers is deepened, and the performance of parameter optimization space is improved. Because the pre-trained ResNet-20 model accepts an image input size of 214 × 214 × 4, we convert each sMRI slice image into a size of 214 × 214 × 4, and then subtract the average intensity value from the corresponding single image channel to normalize the image. Then, we will migrate the ResNet-20 model pre-trained in ImageNet to the data set for fine-tuning.

In addition, because the number of parameters in the fully connected layer is too large to be trained, we removed the original fully connected layer and retrained a fully connected layer *F*(*e*) whose length depends on the number of categories, and *e* represents the number of categories.

In order to prevent over-fitting, we also added a dropout layer with a loss rate of 0.1. *p*_*ave*_ indicates that average pooling is used in this paper. The softmax function used in the training process is shown in the following formula:


(9) $$\begin{array}{l} \text{softmax}\left({\text{F}}_{j}\right)=\frac{\exp^{{\text{F}}_{j}}}{\sum_{k=1}^{e}\exp^{{\text{F}}_{k}}},j=1,2,\cdots \;,e \end{array}$$

We use the cross-entropy loss function to measure the performance of the classification model, and its output is a probability value between 0 and 1. Cross entropy loss increases with the distance that the predicted output deviates from the actual label. If the number of classes is two, the binary cross entropy loss is calculated as follows:


(10) $$\begin{array}{l} L\left(y,p\right)=-\left(y\log p+\left(1-y\right)\log\left(1-p\right)\right) \end{array}$$

If *e* > 2, the classification cross entropy loss is calculated as follows:


(11) $$\begin{array}{l} L\left(y,p\right)=\text{-}\overset{\text{e}}{\underset{\text{c}=\text{1}}{\sum }}{\text{y}}_{\text{o,c}}\log\left(p_{\text{o,c}}\right) \end{array}$$

**Figure 3:**
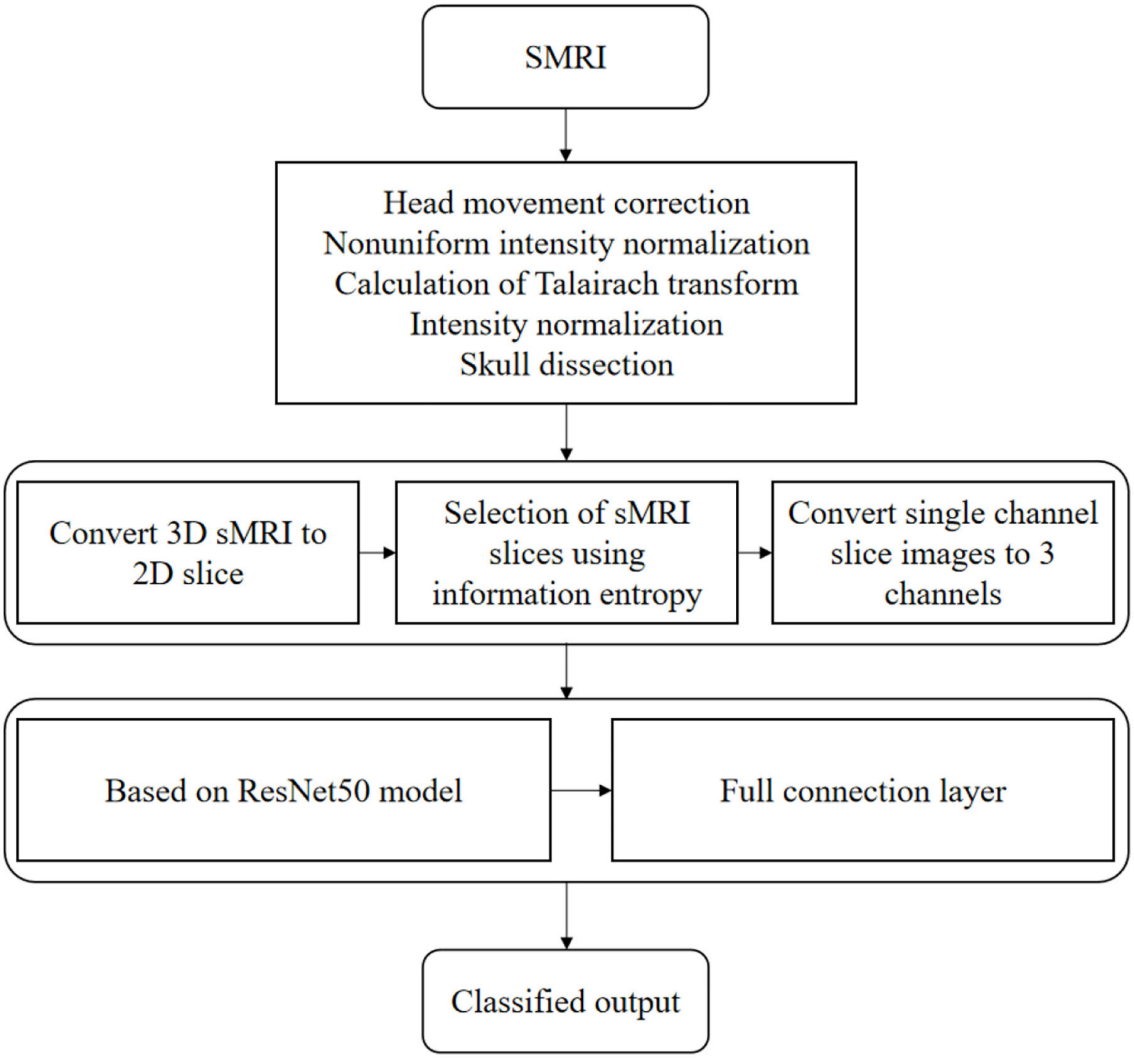
AD classification algorithm flow chart.

Where *y* is the actual value and *p* is the predicted value. The optimization algorithm for updating parameters is RMSprop (root mean square propagation), and the learning rate is set to 0.0001. The flow chart of AD classification algorithm based on transfer learning and deep residual network is shown in Figure 3.

## Result Analysis and Discussion

In this study, k-fold cross-validation method (*K* = 5) is used to evaluate the model, but the problem of this study belongs to classification. For the algorithm proposed in this study, the generalization performance of the model will be evaluated by selecting the functions of calculation accuracy, recall rate, F1 value, AUC value, average validation accuracy and average validation loss. Among them, for the new algorithm proposed in this study and its comparative experiments, all evaluation indexes are used, while for some basic experiments, only verification accuracy is used as the only evaluation index.

### Experimental Results of EEG Feature Extraction Method Based on Multispectral Image Fusion in Multiple Brain Regions

**Figure 4:**
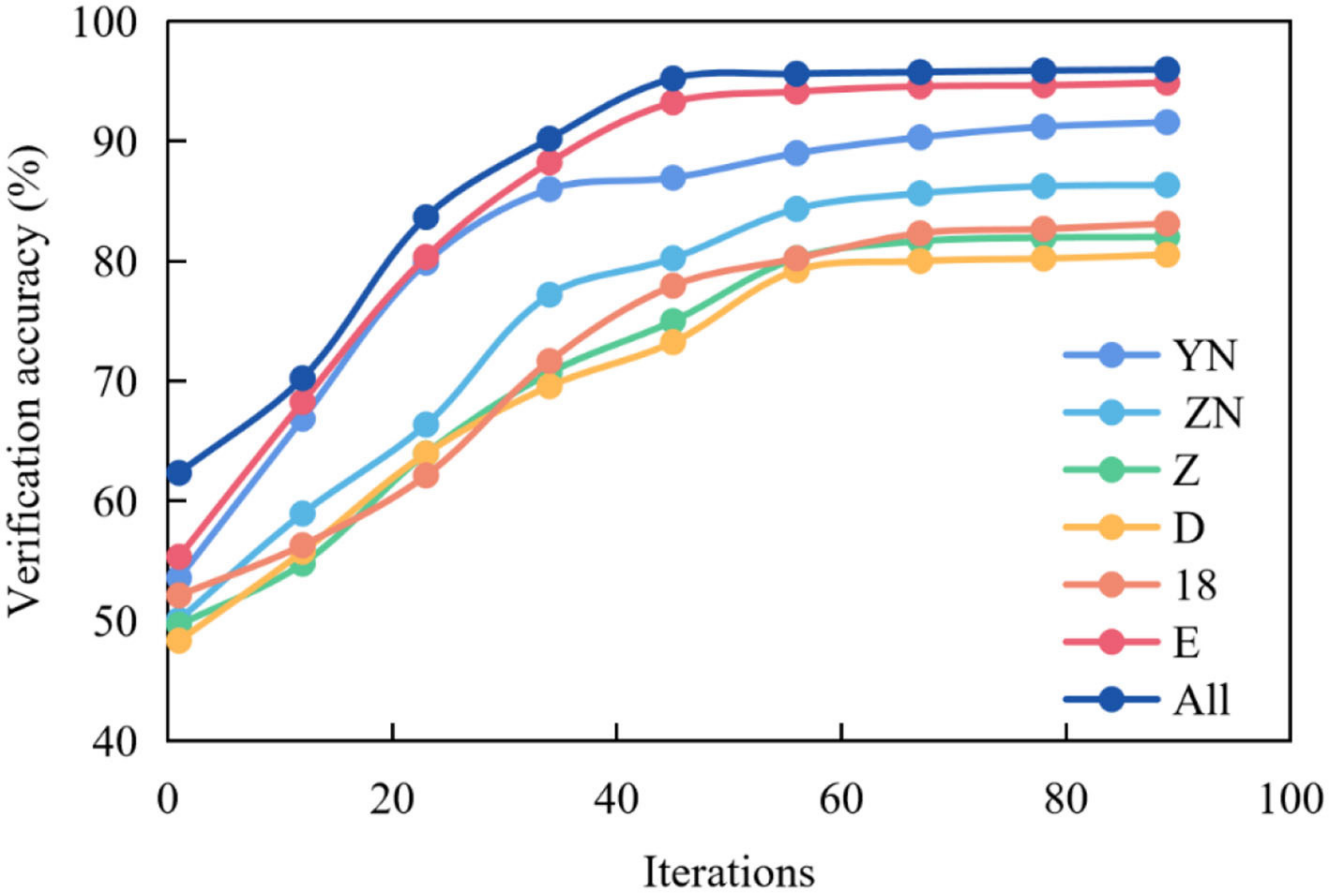
Average verification accuracy curve of multispectral image fusion in multiple brain regions.

Figure 4 shows the graph of the average verification accuracy of multispectral image experiments in multiple brain regions. YN stands for right temporal lobe, ZN stands for left temporal lobe, Z stands for occipital lobe, D stands for parietal lobe, 18 stands for 18 brain regions commonly used in related research, E stands for frontal lobe, and All stands for all brain regions selected by the above five regions.

It can be seen from the figure that the curve representing All is the highest, and the accuracy reaches the highest point around the 30th epochs, which can remain stable, but the curve fluctuates. In other cases, the curves representing E, 18 and D are slightly lower than All, and the accuracy reaches the highest point around the 35th epochs and remains stable. While the curves representing z, ZN and YN are low, and the accuracy reaches the highest point around the 50th epochs and remains stable.

**Figure 5:**
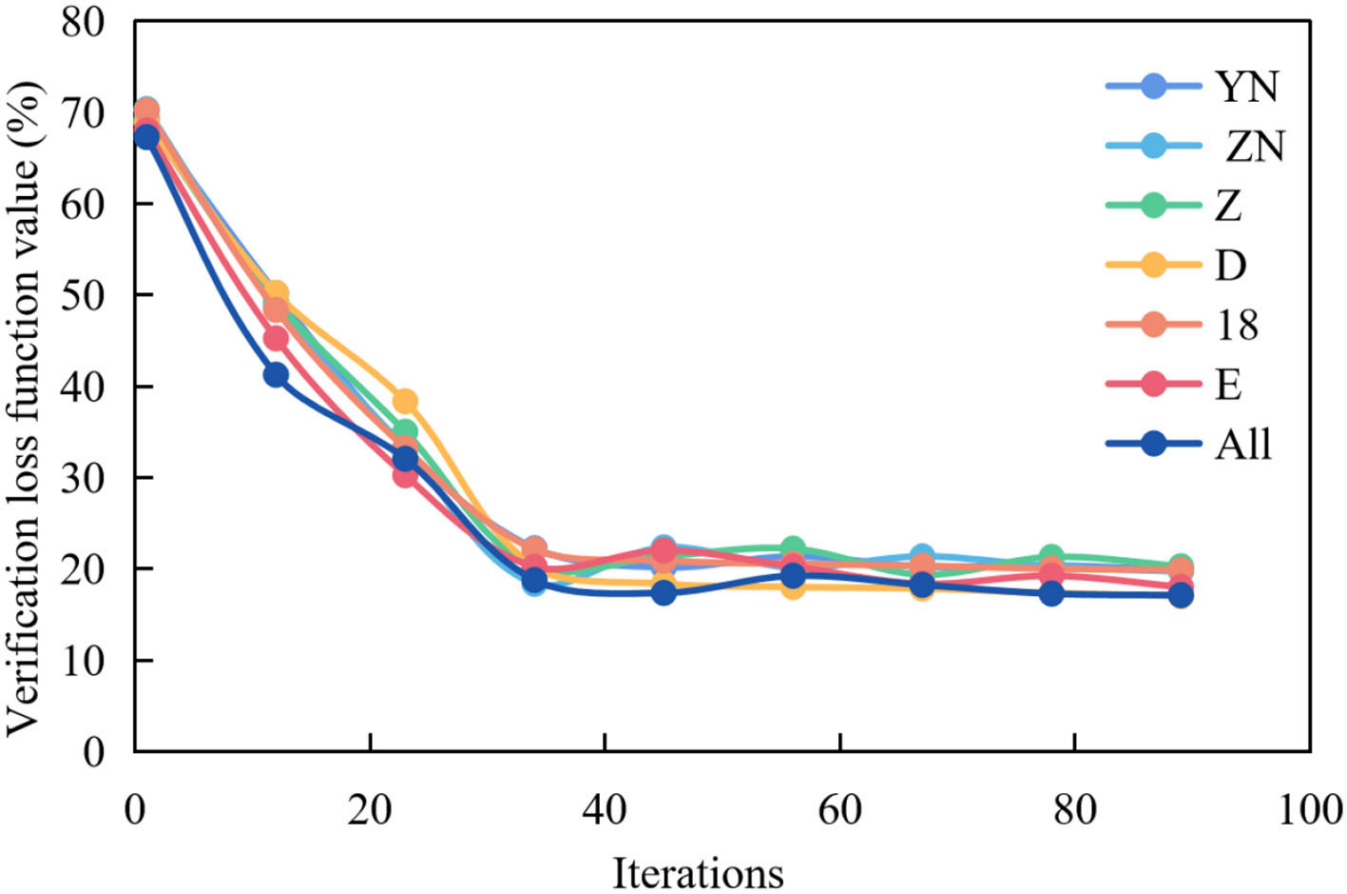
Average verification loss function curve of multispectral image fusion in multiple brain regions.

Figure 5 shows the graph of the average verification loss function of multispectral image experiment in multiple brain regions.

It can be seen from Figure 5 that the curve representing All is the lowest, and the loss function value can reach the lowest around the 30th epochs, but the curve fluctuates greatly. In other cases, the curves representing f, 18 and p are slightly higher than All, and the loss function value reaches the lowest point around the 50th epochs and remains stable. While the curves representing o, LT and RT are higher, and the loss function value reaches the lowest point around the 55th epochs and remains stable.

**Figure 6:**
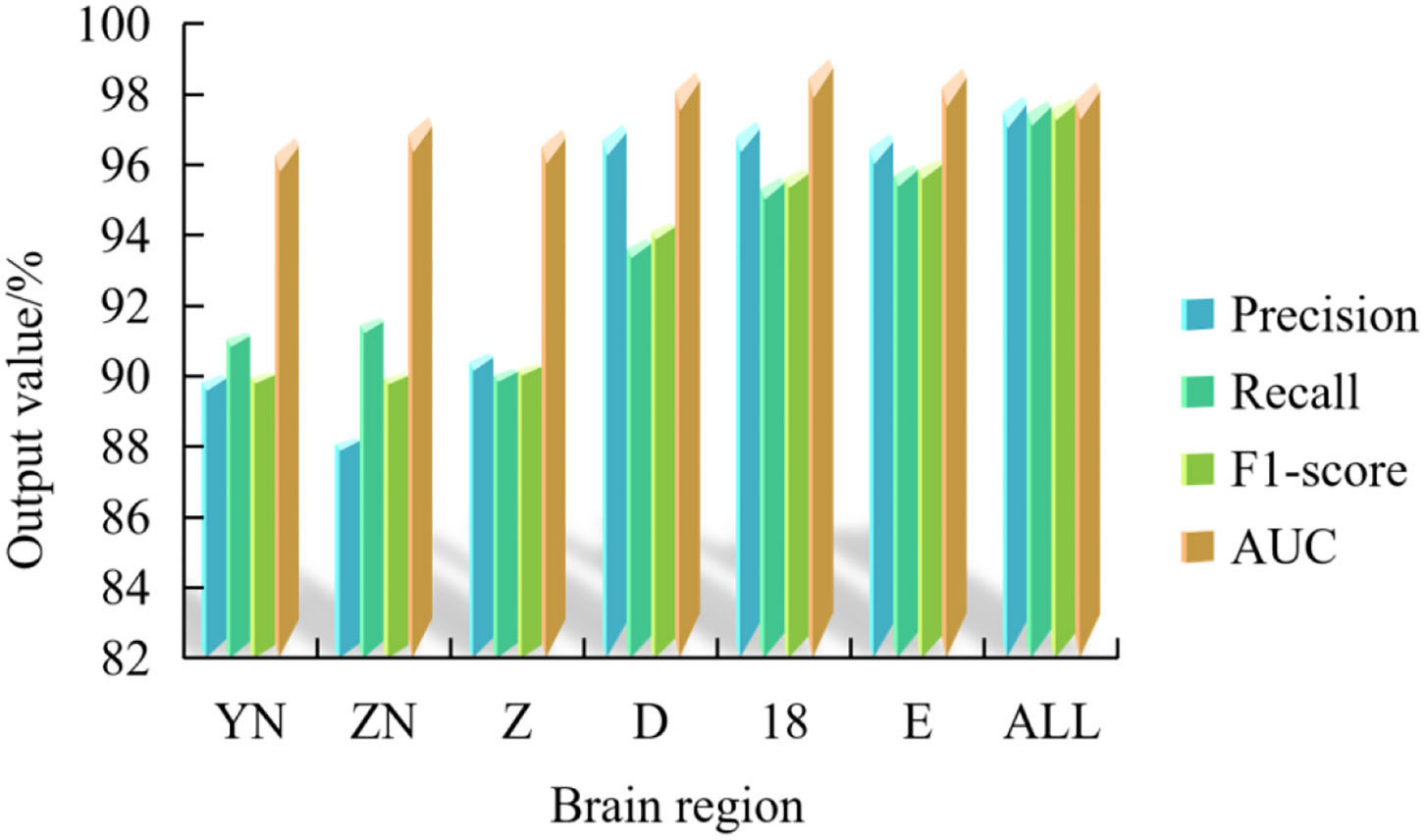
Average accuracy rate, recall rate, F1 value and AUC of multispectral image fusion experiments in multiple brain regions.

Figure 6 shows the average verification accuracy rate, average recall rate, average F1 value and average AUC value of this experiment.

Figure 6 shows that the frontal lobe has the highest value among the 5 multi-spectral image classification performance indexes, which are 97.24% of the average verification accuracy, 97.80% of the average recall rate, 97.60% of the average F1 value and 99.01% of the average AUC value.

In addition, among the seven brain regions, the multispectral image classification performance index of ALL brain regions is in the best position, and the average verification accuracy rate, recall rate, F1 value and AUC value are 97.96, 97.82, 97.88, and 98.35%, respectively.

It can be concluded that the fused multispectral image can complement the information contained in the multispectral image of multi-brain region, so that the fused image has richer feature information, thus improving the classification accuracy. At the same time, the research results of this paper have surpassed the classification performance of previous AMD EEG studies.

In the future, many different image fusion technologies can be used to fuse multispectral images, and the positive and negative relationships among brain regions can also be considered, so as to refine the fusion of multispectral images with multiple brain regions, and the amount of data can be increased by increasing the number of subjects, so as to further verify the rationality of this method.

### AD Classification Algorithm Analysis

All the classification models proposed in this paper are trained and tested in NVIDIA GeForce GTX 1060 GPU system based on Keras. One advantage of Keras is that it can easily and quickly prototype and run seamlessly on GPU. In this paper, transfer learning is introduced, only some layers of the pre-trained ANN model are trained, and the amount of data used in this experiment is small, so this experiment can be completed in this GPU system.

**Figure 7:**
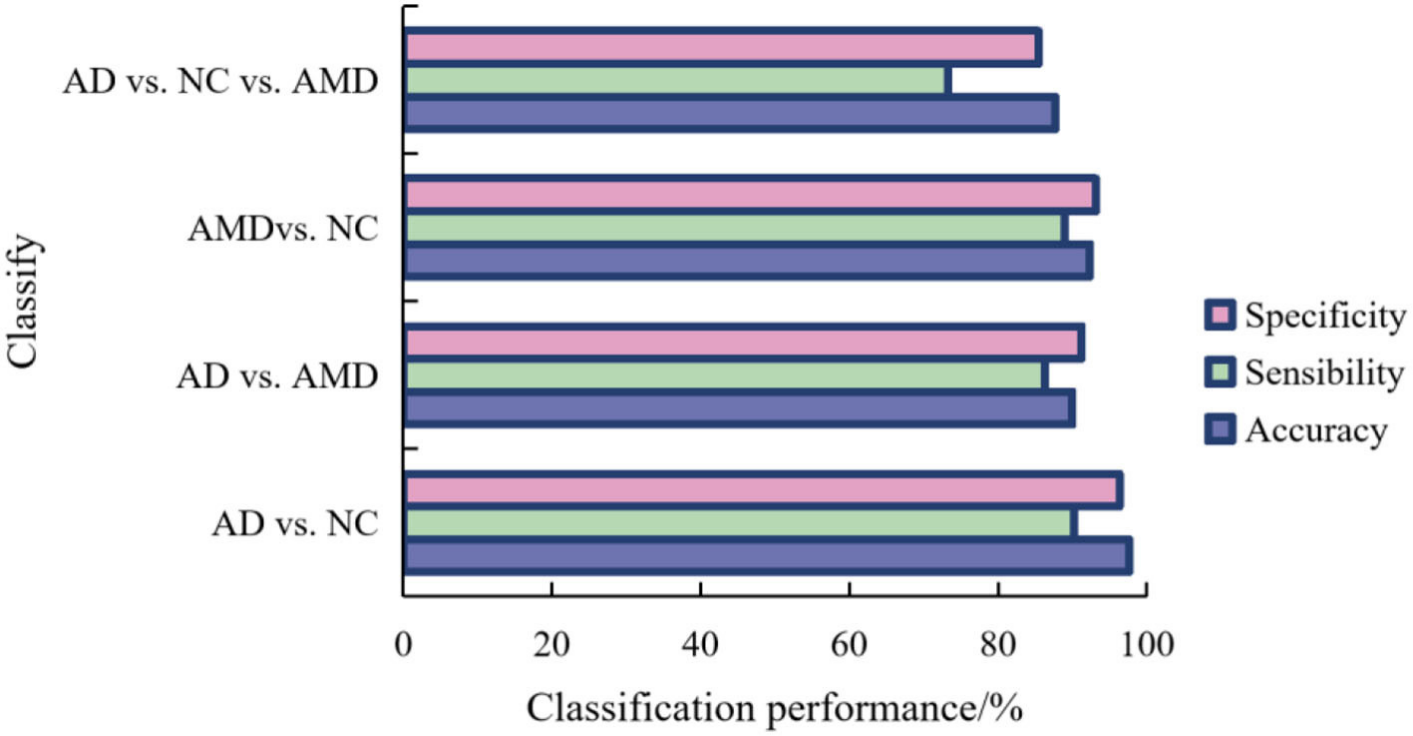
PerformaNCe of ResNet-20_TL model in AD, NC, and AMD classification.

In order to record the experimental results conveniently, we call our method ResNet-20_TL. The classification results of AD, NC, and AMD by the method proposed in this paper are shown in Figure 7.

For binary classification, the accuracy, sensitivity and specificity of the model trained in this paper are 97.66, 90.25, and 96.33% in AD/AMD classification, 90.01, 86.35, and 91.24% in AD/AMD classification, and 92.35 and 82.35% in NC/AMD classification, respectively. For ternary classification, the classification accuracy, sensitivity and specificity of the proposed method are 87.69, 73.21, and 85.46%, respectively.

It can be seen from Figure 7 that AD and NC are the easiest categories to judge, while AMD is easy to be confused with AD and NC. this is because the brain structure of the subjects in different stages of the disease changes to different degrees, the brain tissue of the subjects in AD shrinks seriously, and the brain of the subjects in NC is in a normal state, so the difference in scanned sMRI images is larger, and the proposed method is easier to extract discriminatory features, thus improving the classification performance.

It can also be seen from Figure 7 that compared with binary classification, the accuracy and specificity of AD/NC/AMD classification are lower than 90%, and the sensitivity is lower, mainly because AMD is the intermediate stage between AD and AMD, which leads to the “distance” between features being not obvious enough, so it is not easy to distinguish, which leads to the unsatisfactory accuracy of ternary classification.

In addition, in order to prove the ability of migration learning, we also trained ResNet-20 network from scratch, which is represented by ResNet-20_scratch. For ResNet-20 model trained from scratch, 20 epochs were trained, and the batch was still 64. The adaptive learning rate was used, and the parameters were optimized by RMSProp.

**Figure 8:**
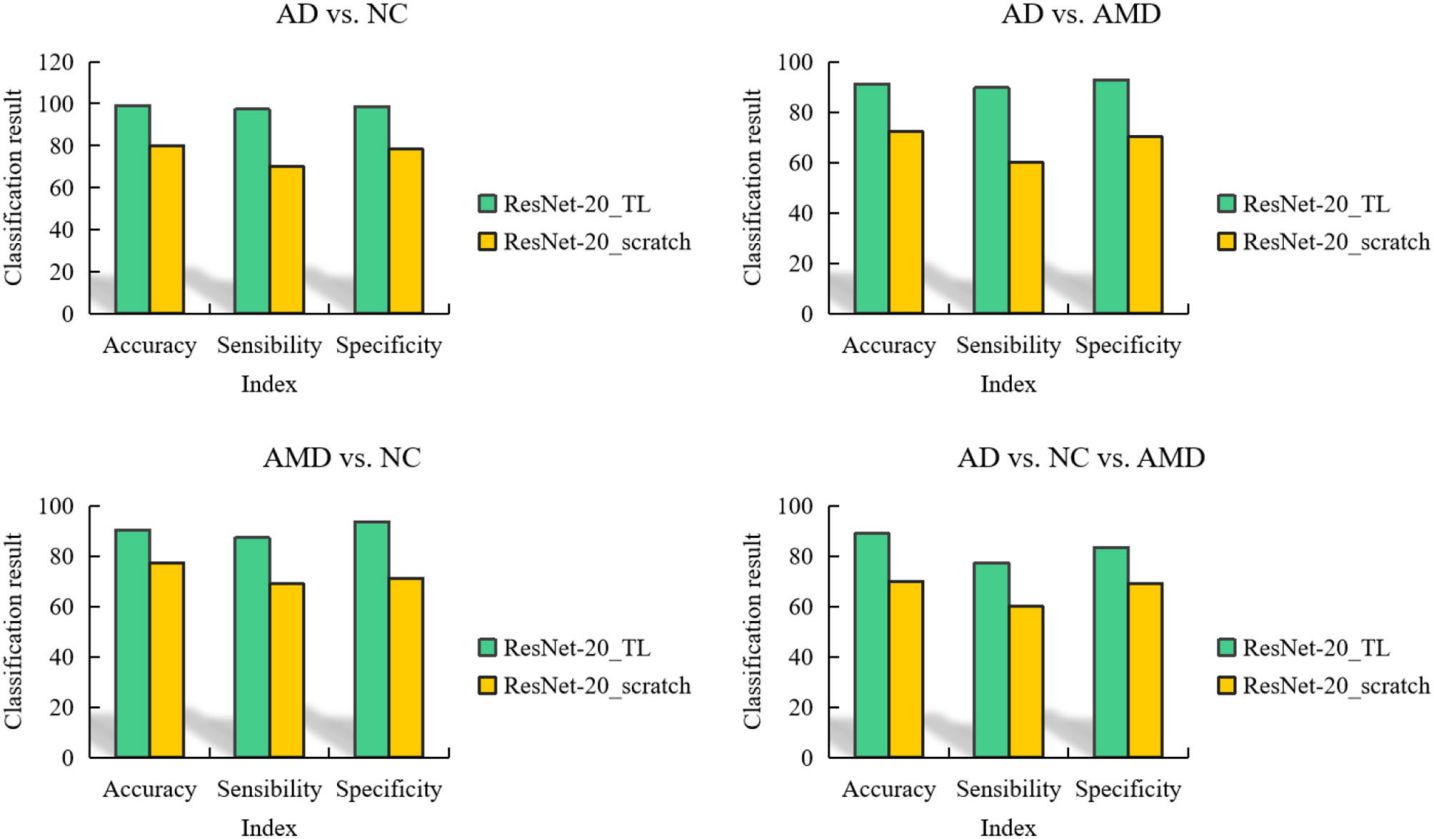
Comparison of classification results obtained by 2 ANN models in 4 classification tasks.

In order to compare the classification performance of ResNet-20_TL model and ResNet-20_scratch model more intuitively, we use a column chart to show it, as shown in Figure 8. Obviously, the fine-tuned ResNet-20 model has better performance in each classification task than the ResNet-20 model trained from scratch.

**Figure 9:**
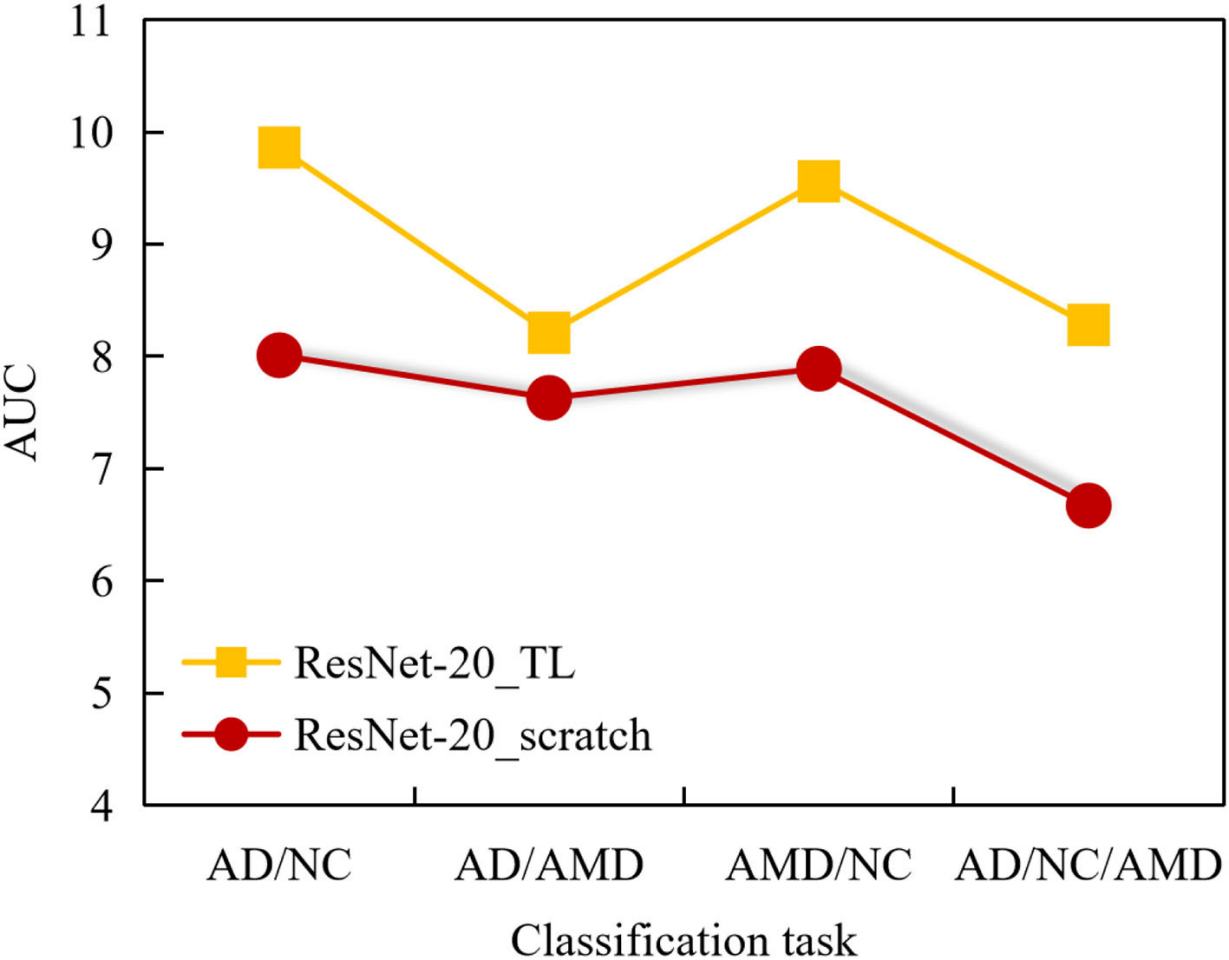
AUC comparison curves of 2 ANN models in classification tasks AMD/NC, AD/NC, AMD/AD, and AD/AMD/NC.

In addition, facing the classification tasks of AMD/NC, AD/NC, AMD/AD, and AD/AMD/NC, the AUC comparison diagram of the area under the curve obtained by the 2 ANN models is shown in Figure 9.

It can be seen from Figure 9 that the AUC value of ResNet-20_scratch model trained from scratch is higher than 0.8 in AD/NC classification, and the AUC value of the other 3 classification tasks is lower than 0.8, and the AUC value of AD/NC/AMD classification is as low as 0.71, which indicates that the auxiliary diagnosis performance of ResNet-20_scratch model is not outstanding enough, and there is room for further improvement.

On the whole, the AUC values obtained by ResNet-20_scratch model in 4 classification tasks are lower than those obtained by ResNet-20_TL model, which also proves the effectiveness of the proposed method. It can be seen from the figure that the AUC of the 4 classification tasks obtained by ResNet-20_TL model is higher than 0.8, and the AUC value of AD/NC classification is up to 0.97, which shows that the ResNet-20_TL model proposed in this paper can help doctors to complete the diagnosis of AD subjects and NC subjects accurately.

## Conclusion

With the increase of AD or AMD in the world, as the early stage of AD, the accurate diagnosis of AMD has become a hot spot in the field of scientific research. In the diagnosis research of AMD, there is still a problem that the classification accuracy is not high enough. ANN has a wide application prospect in many disciplines, such as forensic science. At present, a new upsurge of ANN research is being set off at home and abroad, and new networks with different architectures are constantly coming out, among which BP network, Hopfield network, stochastic neural network, self-organizing neural network, associative memory neural network and CMAC model are more mature. Experiments show that the EEG feature extraction method based on multispectral image fusion in multiple brain regions can obviously improve the classification accuracy of task EEG signals between AMD and NC. Based on the pre-trained ResNet-50 network, the proposed method is evaluated in ADNI data set, and the detailed experimental results are given. The results show that the classification accuracy of the proposed method is better than that of the network model trained from scratch.

In the future, we can consider the positive and negative factors that affect the relationship between brain regions when fusing multispectral images for multiple brain regions, so as to obtain multispectral images that express brain features more accurately. We can try to use 3D convolution neural network to train the above data set.

## Data Availability Statement

The original contributions presented in the study are included in the article/supplementary material, further inquiries can be directed to the corresponding author/s.

## Author Contributions

HY and MZ: conceptualization. YW: methodology and data curation. WM: software and writing—original draft preparation. HY, MZ, and WM: validation. LW: formal analysis and investigation. HY: resources, supervision, project administration, and funding acquisition. MZ: writing—review and editing and visualization. All authors have read and agreed to the published version of the manuscript.

## Funding

This work was supported by the Natural Science Foundation of Heilongjiang Province: Effects of dihydroartemisinin on the prevention and treatment of Alzheimer's disease in mice with age-related macular degeneration (Grant No. LH2021H122) and Basic scientific research Funds of Heilongjiang Provincial undergraduate universities science and technology research project: Study on the association between Alzheimer's disease and age-related macular degeneration based on the down-regulation of Aβ6E10 metabolism by Amylin (Grant No. 2020-KYYWF-0012).

## Conflict of Interest

The authors declare that the research was conducted in the absence of any commercial or financial relationships that could be construed as a potential conflict of interest.

## Publisher's Note

All claims expressed in this article are solely those of the authors and do not necessarily represent those of their affiliated organizations, or those of the publisher, the editors and the reviewers. Any product that may be evaluated in this article, or claim that may be made by its manufacturer, is not guaranteed or endorsed by the publisher.
